# Epidemiology of hepatitis B virus infection: results from a community-based study of 0.15 million residents in South China

**DOI:** 10.1038/srep36186

**Published:** 2016-11-07

**Authors:** Fangfang Zeng, Pi Guo, Yun Huang, Wei Xin, Zhicheng Du, Shuming Zhu, Yu Deng, Dingmei Zhang, Yuantao Hao

**Affiliations:** 1 Department of Medical Statistics and Epidemiology, School of Public Health, Sun Yat-sen University, Guangzhou, Guangdong 510080, China; 2 Department of Preventive Medicine, Shantou University Medical College, Shantou, Guangdong 515041, China; 3 Laboratory of Health Informatics, Guangdong Key Laboratory of Medicine, Sun Yat-sen University, Guangzhou, Guangdong 510080, China

## Abstract

Limited information is available about the current epidemic status of hepatitis B virus (HBV) in Guangdong province in South China, where hepatitis B is endemic. We sought to provide an up-to-date assessment of hepatitis B prevalence in a large population through a community-based study. A total of 169,211 local residents were recruited using the stratified cluster random sampling method from 2014 to 2015, and each participant’s information was collected using an interviewer-administered questionnaire. Accordingly, the prevalence of hepatitis B surface antigen (HBsAg) in the total population was 8.76%. HBsAg prevalence was lowest (0.29%) among children aged 0–12 years and highest (12.71%) among those aged 23–59 years. Moreover, the prevalence (8.82%) in males approximately equalled that (8.65%) in females (*P* > 0.05). Overall, vaccination was effective in preventing HBV infection, regardless of age. Among adults aged 23–59 years, male sex tended to keep the HBsAg persistence. However, reduced persistence for participants with occasional physical exercise and drinking was observed. For participants older than 59 years, a history of prior surgery placed people at high risk for infection. Although Guangdong has successfully decreased the HBsAg prevalence among children, it is urgent to expand vaccination to adults, and employ interventions to reduce the infection risk.

Infection of the hepatitis B virus (HBV) remains a severe public health problem worldwide. There are 2 billion people currently infected with HBV, with 360 million having chronic infections related to HBV and 600,000 deaths annually from either HBV-related liver disease or hepatocellular carcinoma all over the world^1^.

The degree of HBV burden in China is high. According to a 1992 national serosurvey in China, the population’s hepatitis B prevalence was 9.75%, with 120 million Chinese as hepatitis B surface antigen (HBsAg) carriers, 20 million suffering from chronic hepatitis B, and approximately 300,000 deaths each year caused by HBV-related infections^2^,^3^. Liver cancer and cirrhosis have caused high mortality rates in China, and HBV infection is responsible for major deaths from these diseases^4^. Current nucleos(t)ides analogue therapy represents a significant advance in the treatment of chronic hepatitis B, and this kind of therapy is effective in suppressing HBV replication, improving liver function and increasing survival of patients^5^.

In 1992, a nationwide hepatitis B routine immunization was recommended by China’s Ministry of Health, in which parents paid for both the vaccine and the user fee when having their infants vaccinated^6^. To encourage more parents of infants to participate in the immunization campaign, a second attractive policy was introduced in 2002 allowing parents to pay only for the user fee while the vaccine was still freely available. In 2005, both the vaccine and the user fee were waived for the general public. By the year 2006, the prevalence of hepatitis B among people aged 1–59 years had dropped to 7.2%^7^,^8^. The Ministry of Health then issued the “2006–2010 national guidelines for hepatitis B prevention and treatment” to further combat HBV infection^9^.

Studies have shown that relevant risk factors for most HBV infections in developed countries included sexual activity, injectable drug use, a family history of chronic hepatitis B, and occupational exposure^10^,^11^,^12^. Moreover, household contact, vertical transmission haemodialysis, surgical interventions, and receipt of either organs or blood products also resulted in serious HBV infections in developing countries^13^,^14^. In China, the risk factors for HBV infection were male gender, family history of HBV, large family size, and a lack of monogamy^15^,^16^. Overall, unsafe blood transfusion and unprotected sexual contact accounted for most HBV transmission in China^17^. For example, the risk of HBV infection by blood transfusion was estimated to be one in 18,000 in Shenzhen, southern China^18^. The figure was higher than those for Europe and North America which ranged from 1/70000 to 1/130000^19^,^20^. However, most of the previous studies investigating the epidemic of HBV in the Chinese population used limited samples from relatively small regions. Furthermore, there has been a potential shift in hepatitis B infection rates in recent years.

The 1992 serological survey showed that the prevalence of HBsAg in the general population of Guangdong was 17.85%, identifying Guangdong as having one of the highest rates nationwide^21^. In 2002, a descriptive epidemiology study in Guangdong revealed that hepatitis B cases rose gradually and accounted for nearly 79% of total hepatitis cases, and with the highest HBsAg prevalence recorded in the Pearl River Delta region caused by the increased communication resulting from economic development and personnel movement to the region^22^. In 2006, serologic studies of hepatitis B among children in Guangdong province showed that the vaccination had been successfully integrated into routine immunization programmes and was shown to be a cost-effective measure to prevent HBV infection^23^.

Although the immunization programmes have made significant progress in preventing hepatitis B infection, Guangdong still has a severe epidemic of hepatitis B, especially in the opening Pearl River Delta region. However, limited information is available about the current burden status of hepatitis B at the regional population level in the Pearl River Delta. Therefore, to evaluate the current status of hepatitis B burden, as well as to prevent HBV infection by promoting community-based interventions, this study provided an up-to-date assessment of HBsAg prevalence in a large population involving 169,211 residents through a community-based study, and investigated relevant epidemiological factors for HBV infection in the region.

## Methods

### Study population and data collection

This community-based cross-sectional study occurred between January 2014 and December 2015 in two geographically defined regions in the Pearl River Delta of Guangdong province, South China. Stratified cluster random sampling was used to recruit residents from the Pearl River Delta region. The first level of stratification sampling involved cities. Based on the geographic characteristics and the level of socio-economic development, two cities including Guangzhou and Zhongshan (which respectively represent the northern and southern Pearl River Delta) were included in this study. Based on the population density and stability and exposure to certain risk factors, the Liwan and Yuexiu districts from Guangzhou and the municipal district from Zhongshan were chosen. The third level of stratification was the random selection of communities based on the demographic characteristics of each community. Potentially eligible participants within each household in each of 825 communities randomly selected in the study were identified through official residential records to ensure that residents were more widespread throughout the study areas. Invitation letters were delivered door to door by health workers following extensive publicity campaigns.

To encourage participation, all selected residents were informed by local community responsibility doctors, a week before the actual administration. All selected residents were asked to bring their unique national identity cards to be registered in the field. Prior to enrolment, the residents were informed of the details of this study and gave written informed consent for access to their medical records and long-term storage of blood for anonymized medical research purposes.

After informed consent was obtained, all participants were interviewed face-to-face by trained staff in a private examination room. In total, 169,211 participants were recruited in this study. An interviewer-administered questionnaire was used to compile each participant’s demographic information and relevant epidemiological factors for HBV infection. The standard questionnaire consisted of several questions related to general demographic characteristics (age, gender, nationality, career, marital status and education level), cigarette and alcohol consumption frequency, sources of drinking water, physical activity frequency (frequency of taking physical exercise within a certain period of time), migratory status, and medical history (e.g., personal history of surgery, transfusion or trauma, family history of HBV infection, history of hepatitis B vaccination). The physical measurements included height, weight, liver and lung function, blood pressure and pulse rate using standard instruments and protocols.

### Specimen collection and laboratory testing

For each participant, a 5-ml non-fasting venous blood sample (with the time of last meal recorded) was collected aseptically, and the serum was separated from the blood by centrifugation and transported in small vials in an ice-packed box to maintain their temperature at 0–4 °C for a short time period before transport to the laboratory at Da An Gene Company at Sun Yat-Sen University. The serum specimens were screened for HBsAg using enzyme-linked immunoabsorbent assay test kits. The presence of HBsAg was used as an indicator of chronic HBV infection.

All procedures performed in studies involving human participants were in accordance with the ethical standards of the 1964 Helsinki Declaration and its later amendments or comparable ethical standards. The study was approved by the Human Ethics Committee at Sun Yat-Sen University, and written informed consent was obtained from all subjects. Interviews, laboratory tests, and notification of results were provided free to all participants. Test results were enveloped, sealed, and delivered personally to each participant by local community responsibility doctors.

### Statistical analysis

To avoid data entry errors such as mismatches and out-of-range values, all data were input using a double-checking strategy to guarantee the accuracy of the data. Descriptive statistics were used to evaluate the characteristics of the study participants, and the *χ*^2^ test method was employed to compare the categorical variables.

The prevalence of HBsAg by age groups and selected characteristics were calculated separately for the male and female participants and for the full sample. To investigate relevant factors associated with HBV infection, multivariate logistic regressions were performed separately for populations aged 0–22, 23–59, and 60 and above. The dependent variable in the model was the HBV infection status, with an assessment of the HBsAg for each participant; the independent variables included general demographic characteristics and relevant epidemiological factors. Odds ratios (ORs) and 95% confidence intervals (95% CIs) were calculated to assess the association between the selected independent factors and HBV infection.

SAS software version 9.3 (SAS Institute Inc.; Cary, NC) was used for the statistical analyses. All statistical tests were 2-sided, and a *P* < 0.05 was considered to be statistically significant.

### Quality control

Specialists from the Guangdong provincial Centers for Disease Control (CDC), Guangzhou CDC, Guangzhou Information Center for Health, and Sun Yat-sen University convened several times to discuss the design of the study protocol, statistical issue, and project implementation of this study. All staff was trained to conduct interviews, process blood specimens and perform laboratory testing uniformly. To enhance the quality of the study, a pilot field study was performed with repeat questionnaire and measures on selected items on participants who were randomly selected from the studied communities. Both of the pilot field study and the actual survey were conducted according to the standard field survey operating procedures.

## Results

### Baseline characteristics of study participants

A total of 169,211 participants completed the questionnaires and blood samples collection, and included in the analysis after checking the validity of each questionnaire.

Table 1 shows the baseline characteristics of the study participants. Among the study population, 49.58% of the participants were aged 23–59 years. The male to female ratio was 0.47:1, and 97.66% were Han Chinese. Most of the participants (44.17%) had the height less than 155 cm, whereas 21.77% of individuals had a height between 155 cm to 159 cm. Moreover, a high percentage had a BMI ranging from 18.5 kg/m^2^ to 22.5 kg/m^2^, accounting for 45.23% of the total population. Among the study participants, 64.58% had primary and middle school education degrees, and married individuals composed the largest percentage (78.27%) of the population. The percentage of participants who received hepatitis B vaccination was significantly higher among children less than 23 years old (90.63%) than among adults aged 23–59 years (17.71%) and 60+ years old (13.70%).

### Prevalence of HBsAg by demographic characteristics

**Figure 1:**
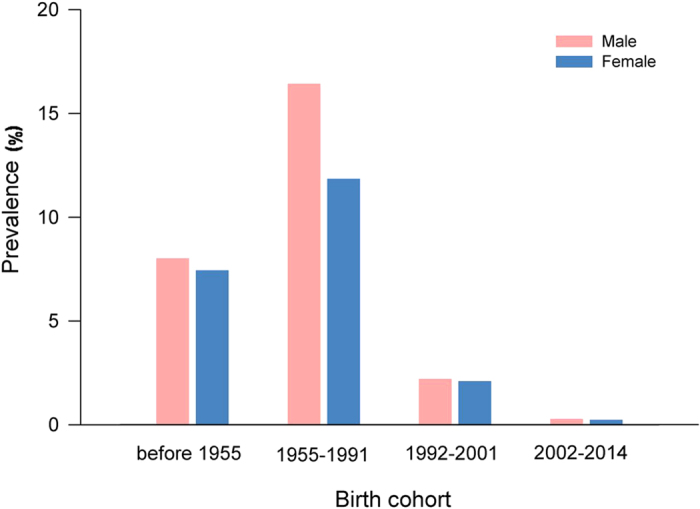
Prevalence of hepatitis B surface antigen (HBsAg) among the study participants by gender according to different birth cohorts. This descriptive analysis was performed according to four birth cohorts: the participants born before 1955, during 1955–1991, 1992–2001, and 2002–2014, respectively.

**Figure 2:**
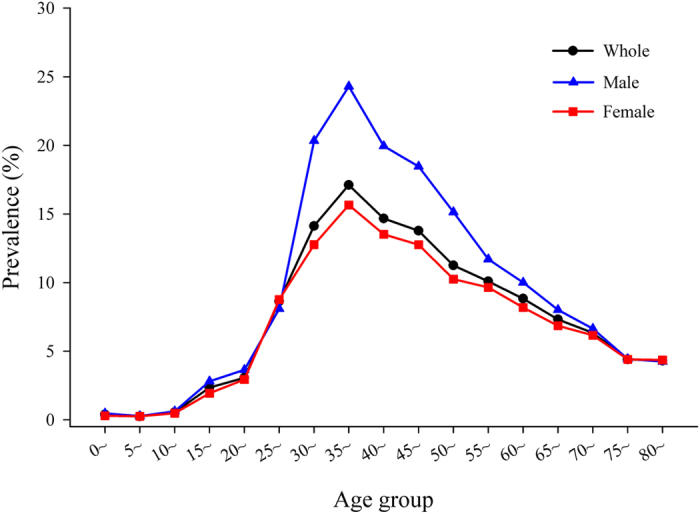
Prevalence of hepatitis B surface antigen (HBsAg) among the study participants by gender and age group. This descriptive analysis was performed according to three groups of people: the whole group of participants enrolled in this study, the male group of participants and the female group of participants.

Overall, the HBsAg prevalence of the study population was 8.76%. Table 2 shows the HBsAg prevalence based on the selected characteristics of the study participants. Accordingly, the prevalence was lowest among children aged 0–12 years old (0.29%) and highest among those aged 23–59 years (12.71%). We also calculated the HBsAg prevalence by gender according to different birth cohorts, and found that the prevalence was significantly favourable for both genders among successive birth cohorts born in 1992 (Fig. 1). We further investigated the prevalence variance by age group every 5 years for the full, male and female populations separately (Fig. 2). It was obvious that, regardless of gender, the prevalence of HBsAg was highest among those aged 35–39 years (17.71% for the total population, 25.58% for males and 16.27% for females), whereas the prevalence was lowest among children aged 5–9 years old (0.27% for the total population, 0.27% for males and 0.26% for females).

In Table 2, children less than 22 years old had a significantly lower prevalence of HBsAg than the other two defined age groups. The HBsAg prevalence (8.82%) of males was slightly higher than that (8.65%) of females, but the difference was not statistically significant (*P* > 0.05). Based on the stratification analysis, similar patterns of HBsAg prevalence among males and females were observed for most age groups (Fig. 2). The prevalence in the minority population was higher than that in the Han population (9.88% *vs*. 8.74%, *P* < 0.05). Among participants older than 23 years of age, people with only a primary school education had the highest prevalence among the participant with different levels of education. Similarly, for participants older than 23 years of age, the HBsAg prevalence was highest among agricultural, forestry and fishery producers. Among the participants aged 23–59 years, married people had the highest prevalence of HBsAg (12.98%); however, the highest percentage (8.18%) for individuals aged 60+ years was observed among divorcees. This study consistently found that vaccinated participants had a lower prevalence than unvaccinated participants among the following age groups: 0–22 years (0.35% *vs*. 2.50%, *P* < 0.05), 23–59 years (7.10% *vs*. 14.85%, *P* < 0.05) and 60+ years (5.04% *vs*. 8.25%, *P* < 0.05), respectively.

**Figure 3:**
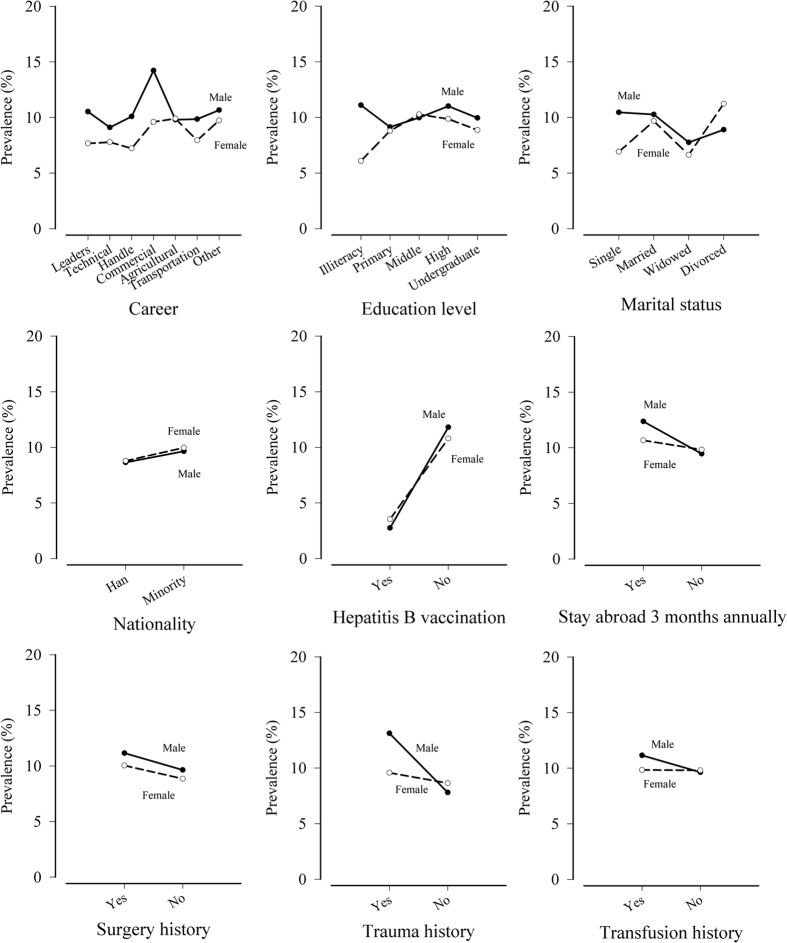
Prevalence of hepatitis B surface antigen (HBsAg) of the selected characteristics by gender. The selected characteristics of participants included career, education level, marital status, nationality, stay abroad more than three months annually, and individual history of hepatitis B vaccination, surgery, trauma and transfusion.

The HBsAg prevalence by gender according to the selected characteristics is shown in Fig. 3. For most careers and education levels, the prevalence in males was higher than that in females. Among single, married and widowed participants, males had a higher prevalence than females. However, divorced males tended to have a lower prevalence than divorced females. The HBsAg prevalence in males and females was similar for different types of nationalities. Among the study participants, people with an individual history of surgery, trauma or transfusion tended to have a higher prevalence than those without such a history, regardless of gender. A similar pattern was observed in participants who stayed abroad longer than three months annually in contrast with those who did not. Moreover, people who were vaccinated for hepatitis B had a lower prevalence than the unvaccinated, regardless of gender.

### Relevant factors associated with hepatitis B infection

The relationships between hepatitis B infection and relevant epidemiological factors based on multivariate logistic regression analysis according to different age groups were reported (Table 3). Among the 0–22 age group, the only significant factor associated with hepatitis B infection was an individual history of hepatitis B vaccination. After adjusting for all relevant factors, vaccination was associated with a diminished risk of infection (OR: 0.10, 95% CI: 0.04 to 0.24).

For participants aged 23–59 years, the factor of getting vaccination was shown to decrease the risk of hepatitis B infection (OR: 0.56, 95% CI: 0.43 to 0.73). Male sex (OR: 1.51, 95% CI: 1.20 to 1.91) tended to keep the HBsAg persistence in the study population. In addition, reduced persistence for participants with occasional alcohol consumption (OR: 0.62, 95% CI: 0.41 to 0.93) and occasional physical exercise (OR: 0.64, 95% CI: 0.49 to 0.84) were observed.

For adults aged 60 years and older, vaccination positive vaccination status was determined to be a protective factor against hepatitis B infection (OR: 0.51, 95% CI: 0.32 to 0.82). In addition, having any surgical history tended to increase the probability of hepatitis B infection (OR: 1.52, 95% CI: 1.10 to 2.10) for this age group.

## Discussion

This study provided an up-to-date assessment of hepatitis B infection and relevant factors among community residents in the Pearl River Delta region during the period of 2014–2015. To our knowledge, this is the largest community-based study ever conducted in South China. Overall, this study reports that the prevalence of HBsAg in the general population in the study region was 8.76%.

Actually, in 1992 the World Health Organization (WHO) recommended the integration of hepatitis B vaccine into the national immunization programs in all countries of high HBV endemic rate^24^. In response, China’s Ministry of Health started to conduct a program of hepatitis B routine immunization nationwide that year. Over the past 25 years, there were two main policies successively promoting the integration of hepatitis B vaccine into routine infant immunization in China, occurring in two crucial calendar years of 1992 and 2002. By far, the hepatitis B vaccine has been fully integrated into routine infant immunization. In order to evaluate the impact of the hepatitis B vaccination program in Guangdong province of South China, we grouped the study participants into four groups with the cut-offs of age set as 0~12, 13~22, 23~59, and over than 60 years old. Thus, we generated four birth cohorts for analysis: participants born during 2002 to 2014, 1992 to 2001, 1955 to 1991, and before 1955. We measured the HBsAg prevalence of residents via an updated hepatitis B serosurvey, analyzed the prevalence and the relevant factors according to different age groups, and finally provided the evidence on the importance and the urgent request of HBV immunizations for policy making of the government.

In fact, the population in Guangdong province experienced a significant decline in HBsAg prevalence from 16.67% to 11.10% between 1992 and 2006, with an overall decrease of nearly 5.6%^7^,^21^. In this work, we found that the prevalence of HBsAg in the entire population was 8.76% during the study period. It is obvious that Guangdong made significant progress in the prevention of hepatitis B. However, when moting that the national prevalence of HBsAg decreased from 9.8% in 1992 to 7.2% in 2006^2^,^7^, Guangdong province leads in prevalence nationwide and continues to suffer from this major public health problem among local residents.

This study showed that children aged 0–12 and 13–22 years old had the lowest HBsAg prevalence (0.29% and 2.24%, respectively) compared with the other two age groups. In particular, the HBsAg prevalence of 0.27% was lowest among children aged 5–9 years old according to the age subgroup analyses. The findings in this study indicated that there was a significant decline in hepatitis B epidemics among children when compared with previous reports^21^,^22^,^23^. First, this transformation occurred after the hepatitis B vaccine was recommended as part of routine infant immunizations in 1992, which shifted children to a relatively low-risk group. Second, we affirmed that in 2002, after China integrated the hepatitis B vaccine into the expanded routine immunization programme (with an emphasis on providing a timely dose of the vaccine within 24 hours following the child’s birth) to prevent hepatitis B infection, there was another downward trend in HBsAg prevalence in children. In addition, the highest HBsAg prevalence in 2002 and 2006 occurred in adults aged 20–24; however, the highest prevalence appeared in adults aged 35–39 years old during the study period. The 15-years delay in this peak may be attributed to a 22-year effort of successive vaccination programmes in China and the accelerated progress of the national guidelines for hepatitis B prevention and treatment. Overall, the study region had accomplished the goal of reducing the HBsAg prevalence rate to less than 1% for children less than 5 years of age^25^,^26^.

In 1992 and 2002, the HBsAg prevalence rates for males in Guangdong were 18.82% and 15.99%, respectively, which are significantly higher than the rates for females (14.81% and 10.36%, respectively) (both *P* < 0.05)^7^. However, in 2006, there was no significant difference between the HBsAg prevalence in males and females (11.96% *vs*. 10.33%, *P* > 0.05)^25^. This result is consistent with the finding in this work and supports our speculation that the current prevalence of HBsAg is similar between genders. Based on these comparisons, we also observed great progress in diminishing the risk of hepatitis B infection for both genders, with a large reduction in HBsAg prevalence for males but a relatively small decrease for females. The finding was similar to the result from a study conducted in Zhejiang province in 2007^27^. This finding may be related to the vigorous scientific publicity surrounding hepatitis B knowledge which promoted awareness of the need for personal protection among men and improved hygiene and sanitation practices in Guangdong province^25^.

Furthermore, this work determined the relevant factors for HBV infection among the study population. It revealed that for those in the 0–22 age group, received the hepatitis B vaccination was only associated with the decreasing risk of hepatitis B infection. A similar result was also observed for the other two age groups in this study. The findings presented the importance of the inclusion of the hepatitis B in routine vaccine immunization programmes conducted in China, especially for people born after 1992. For participants aged 23–59 years old, relevant factors including female gender, getting vaccination, occasional alcohol intake and occasional physical exercise were identified. In contrast, the individual history of getting vaccination and surgery were significant among adults aged above 60 years old.

Overall, there were some differences in the associated factors of HBV infection among different birth cohorts. Among the subgroup of population aged 23–59 years old, male sex tended to keep the HBsAg persistence in the study population. Some previous studies showed important relationship between HBV infection and other sexually transmitted disease, especially for the lack of protection awareness in sex activities for men^28^,^29^,^30^. However, there was no HBsAg carriage data about the partner of the infected male patients in this study. So the relationship between HBV infection and unprotected sex activities could not be determined herein. With regard to physical activity, our finding suggested that sufficient of physical exercise would improve liver function and diminished the HBsAg persistence, which was consistent with a previous study^31^. The factor of an individual history of surgery turned out to be significantly associated with HBV infection in people aged over 60, whereas it did not appear in the other two age groups of people. It showed a more favourable situation in Guangdong than other regions such as Anhui and Taiwan^32^,^33^. The finding suggested that unsafe blood transfusion during surgery might be mainly responsible for the prevalence among adults aged 60+ years old. Guangzhou is a large blood collection and supply centre in China with a high frequency of population mobility, and this may strongly contribute to the increased blood transmission risk. However, to decrease the risk of HBV blood transmission, the government has taken a series of positive actions, and the risk of infection had reduced after this strategy to some extent^34^. In fact, health-care-related transmission has long been known as an important source of HBV infections. Accordingly, during a surgery there were three possible transmission routes for infection including from a surgeon to a patient, from contaminated surgical instruments to a patient, and from an HBV-positive patient to another patient in the same hospital room^35^,^36^,^37^. So we should also paid attention on the prevention of the health-care-related transmissions of HBV in the study area.

In addition, transmission via sexual behavior was significantly associated with the acute HBV infection among adults in some areas in China^19^,^38^. Characteristics including marital status, education level, and career type were expected to affect HBV infection^7^,^39^,^40^. It may be due to different socio-economic status with unequal access to medical care resource such as vaccinations against hepatitis B^7^. However, statistically significant correlations were not found between these potential factors and HBV infection based on the multivariate analysis herein. More studies should be conducted to investigate the associations of these factors for individuals residing in the Pearl River Delta region.

On the whole, although the HBsAg prevalence had sharply decreased among the vaccinated people, there were still a relatively large number of individuals carried HBsAg in Guangdong. The coverage of vaccination in the study area still demands an effective expansion. To fully protect the whole population, it is necessary to expand the vaccination programs to more children and the high-risk adults. The government should enhance the implementation of hepatitis B vaccination programs and warrant the quality of vaccines.

Several limitations of our study should be mentioned. First, in this study the participants were limited to community residents who had official residential records. This might unintentionally exclude the short-term floating population, rural residents and unregistered children born outside of family planning, and not accurately estimate overall HBsAg prevalence in the study area. Second, we could not better analyze the item of career and provide more information due to the missing data on career in this study.

In conclusion, Guangdong has successfully integrated the hepatitis B vaccine into routine immunization programmes and achieved significant progress in decreasing HBsAg prevalence among children born after 1992. These efforts should be sustained to make a greater progress in the future. To achieve the national goal of decreasing the prevalence of HBsAg of the entire population to less than 7%, free immunization for infants should be implemented strictly, and there is an emergent demand to expand vaccination to adults, including migrants and minority populations. Moreover, the regulation, technology and management of blood transfusion should be improved to decrease the risk of HBV infection in high-risk areas. Community-based health education should be available to promote residents’ awareness of secure sexual behaviour and blood donation as well as encourage people to develop healthy life habits such as regular physical exercise.

## Additional Information

**How to cite this article**: Zeng, F. *et al*. Epidemiology of hepatitis B virus infection: results from a community-based study of 0.15 million residents in South China. *Sci. Rep.* **6**, 36186; doi: 10.1038/srep36186 (2016).

**Publisher’s note**: Springer Nature remains neutral with regard to jurisdictional claims in published maps and institutional affiliations.

## Figures and Tables

**Table 1:** Characteristics of the study participants in the Guangdong province of South China during 2014–2015.

Characteristics	Category	Frequency	Percentage
Age (years)	0–12	16188	11.63
	13–22	2904	2.08
	23–59	69026	49.58
	60+	51103	36.71
Gender	Male	53718	31.75
	Female	115493	68.25
Height (cm)	<155	70051	44.17
	155–159	34533	21.77
	160–164	25936	16.35
	≥165	28071	17.70
BMI (kg/m^2^)	<18.5	16104	10.18
	18.5 to <22.5	71553	45.23
	22.5 to <25	51934	32.83
	25 to <30	15758	9.96
	≥30	2849	1.80
Nationality	Han	164425	97.66
	Minority	3946	2.34
Educational level	Illiteracy	7305	4.87
	Primary school	56573	37.68
	Middle school	40384	26.90
	High and vocational school	34681	23.10
	Undergraduate and above	11208	7.46
Career	Leaders of enterprise unit	2272	1.50
	Technical personnel	4508	2.98
	Handle affairs personnel	3851	2.55
	Commercial personnel	6885	4.55
	Agricultural, forestry and fishery producers	25948	17.16
	Transportation equipment operators	7553	5.00
	Others	100179	66.26
Marital status	Single	24254	15.77
	Married	120380	78.27
	Widowed	7719	5.02
	Divorced	1451	0.94
Hepatitis B vaccination (aged 0–22 years)	Yes	5457	90.63
	No	564	9.37
Hepatitis B vaccination (aged 23–59 years)	Yes	1886	17.71
	No	8763	82.29
Hepatitis B vaccination (aged 60+ years)	Yes	6997	13.70
	No	44082	86.30

Two basic descriptive statistics including the frequency and percentage were used.

**Table 2:** Prevalence of hepatitis B surface antigen (HBsAg) according to the selected characteristics.

Characteristics	Frequency	Prevalence (%)	*P*
Age (years)			<0.0001
*0*–*12*	16188	0.29	
*13*–*22*	2904	2.24	
*23*–*59*	69026	12.71	
*60+*	51103	7.33	
Gender			0.2243
*Male*	53718	8.82	
*Female*	115493	8.65	
Nationality			<0.0001
*Han*	164425	8.74	
*Minority*	3946	9.88	
Educational level (aged 23–59 years)			<0.0001
*Illiteracy*	743	10.50	
*Primary school*	11288	13.25	
*Middle school*	19241	13.07	
*High and vocational school*	20059	12.53	
*Undergraduate and above*	6974	11.03	
Educational level (aged 60+ years)			0.0002
*Illiteracy*	3504	6.08	
*Primary school*	13956	7.48	
*Middle school*	17966	7.22	
*High and vocational school*	11973	6.92	
*Undergraduate and above*	3312	5.98	
Career (aged 23–59 years)			<0.0001
*Leaders of enterprise unit*	980	12.14	
*Technical personnel*	2214	10.98	
*Handle affairs personnel*	2115	10.07	
*Commercial personnel*	4629	12.66	
*Agricultural*, *forestry and fishery producers*	8158	13.89	
*Transportation equipment operators*	2639	12.62	
*Others*	41596	12.97	
Career (aged 60+ years)			<0.0001
*Leaders of enterprise unit*	1130	5.93	
*Technical personnel*	1969	5.33	
*Handle affairs personnel*	1527	5.44	
*Commercial personnel*	1893	6.71	
*Agricultural*, *forestry and fishery producers*	15767	7.83	
*Transportation equipment operators*	4558	6.38	
*Others*	23144	7.37	
Marital status (aged 23–59 years)			<0.0001
*Single*	5168	10.20	
*Married*	51452	12.98	
*Widowed*	1046	11.57	
*Divorced*	904	12.06	
Marital status (aged 60+ years)			<0.0001
*Single*	1308	5.73	
*Married*	40900	7.37	
*Widowed*	6569	6.00	
*Divorced*	501	8.18	
Hepatitis B vaccination (aged 0–22 years)			<0.0001
*Yes*	17295	0.35	
*No*	1792	2.50	
Hepatitis B vaccination (aged 23–59 years)			<0.0001
*Yes*	56777	7.10	
*No*	12220	14.85	
Hepatitis B vaccination (aged 60+ years)			0.0003
*Yes*	6997	5.04	
*No*	44082	8.25	

Characteristics of the study participants included age, gender, nationality, education level, career, marital status, and individual history of hepatitis B vaccination. Data were expressed as the frequency and prevalence, and Chi-square test was performed to compare the differences among different groups. The statistical significance level of *P* < 0.05 was used.

**Table 3:** Relevant factors associated with hepatitis B infection among participants according to different age groups (0–22, 23–59 and 60+ years) using multivariate logistic regression models.

Age group	Variable	Category	OR	95% CI for OR		*P*
				Lower	Upper	
0–22†	Hepatitis B vaccination	No*	1.00			
		Yes	0.10	0.04	0.24	<0.0001
23–59‡	Hepatitis B vaccination	No*	1.00			
		Yes	0.56	0.43	0.73	0.0054
	Gender	Female*	1.00			
		Male	1.51	1.20	1.91	0.0045
	Education level	Illiteracy*	1.00			
		Primary school	0.69	0.26	1.83	0.4563
		Middle school	1.12	0.46	2.70	0.8080
		High and vocational school	0.90	0.38	2.17	0.8190
		Undergraduate and above	0.67	0.27	1.64	0.3763
	Alcohol consumption	Never*	1.00			
		Occasionally	0.62	0.41	0.93	0.0221
		Often	0.18	0.03	1.32	0.0915
		Every day	0.87	0.19	3.99	0.8593
	Physical activity frequency	Never*	1.00			
		Occasionally	0.64	0.49	0.84	0.0011
		More than once per week	0.91	0.69	1.19	0.4849
		Every day	0.83	0.63	1.08	0.1684
60+§	Hepatitis B vaccination	No*	1.00			
		Yes	0.51	0.32	0.82	0.0057
	History of surgery	No*	1.00			
		Yes	1.52	1.10	2.10	0.0112
	Career	Leaders of enterprise unit*	1.00			
		Technical personnel	1.12	0.48	2.59	0.7941
		Handle affairs personnel	0.79	0.31	2.01	0.6237
		Commercial personnel	1.46	0.62	3.44	0.3845
		Agricultural, forestry and fishery producers	2.25	0.64	7.89	0.2051
		Transportation equipment operators	0.67	0.26	1.69	0.3951
		Others	1.59	0.77	3.27	0.2104

Odds ratio (OR) and its 95% confidence interval (95% CI) were calculated to assess the association between HBV infection and relevant factors. The statistical significance level of *P* < 0.05 was used.

^†^Adjusted variables: gender, BMI, nationality, history of surgery, history of trauma, history of transfusion, history of having genetic disease, and drinking water.

^‡^Adjusted variables: BMI, nationality, career, history of surgery, history of trauma, history of transfusion, history of having genetic disease, drinking water, stay abroad more than three months per year, cigarette and alcohol consumption frequency.

^§^Adjusted variables: gender, education level, marital status, physical activity frequency, BMI, nationality, history of trauma, history of transfusion, history of having genetic disease, drinking water, stay abroad more than three months per year, cigarette and alcohol consumption frequency.

^*^Reference category.
